# Supplementary material for: Design and Validation of qPCR-Specific Primers for Quantification of the Marketed Terfezia claveryi and Terfezia crassiverrucosa in Soil
Source: J Fungi (Basel). 2022 Oct 17;8(10):1095. doi: 10.3390/jof8101095 (PMC9605127; doi:10.3390/jof8101095)
Supplement: Supplementary file 1 [file jof-08-01095-s001.zip › jof-1951950-supplementary.pdf]

Table S1. Accession number from GenBank and RefSeq databases (NCBI) of the species used for *turmas* primers design.

Accession No.	Species	Accession No.	Species
AF387652.1	*Picoa lefebvrei*	KP728826.1	*Terfezia cistophila*
GQ981519.1	*Picoa lefebvrei*	KP728827.1	*Terfezia cistophila*
JN392146.1	*Picoa lefebvrei*	KP728829.1	*Terfezia cistophila*
KR073956.1	*Picoa juniperi*	NR_160445.1	*Terfezia cistophila*
KR073959.1	*Picoa juniperi*	HQ698076.1	*Terfezia claveryi*
FM206445.1	*Geopora arenicola*	HQ698078.1	*Terfezia claveryi*
FM206448.1	*Geopora arenicola*	HQ698081.1	*Terfezia claveryi*
AF387649.1	*Geopora cooperi*	HQ698084.1	*Terfezia claveryi*
JF908023.1	*Geopora cooperi*	MF940185.1	*Terfezia claveryi*
MK446225.1	*Geopora cooperi*	MF940191.1	*Terfezia claveryi*
MK359194.1	*Geopora cooperi*	MF940196.1	*Terfezia claveryi*
MG949282.1	*Tirmania honrubiae*	MF940199.1	*Terfezia claveryi*
MG949283.1	*Tirmania honrubiae*	MF940202.1	*Terfezia crassiverrucosa*
MG949284.1	*Tirmania honrubiae*	MF940203.1	*Terfezia crassiverrucosa*
MG949285.1	*Tirmania honrubiae*	MH810272.1	*Terfezia crassiverrucosa*
MG949286.1	*Tirmania honrubiae*	NR_159052.1	*Terfezia crassiverrucosa*
MG949287.1	*Tirmania honrubiae*	MN438323.1	*Terfezia dunensis*
MG949289.1	*Tirmania honrubiae*	MN438324.1	*Terfezia dunensis*
MG949288.1	*Tirmania honrubiae*	MN438325.1	*Terfezia dunensis*
NR_164270.1	*Tirmania honrubiae*	NR_169982.1	*Terfezia dunensis*
AF276665.1	*Tirmania nivea*	HM056205.1	*Terfezia eliocrocae*
AF276666.1	*Tirmania nivea*	HM056206.1	*Terfezia eliocrocae*
AF276667.1	*Tirmania nivea*	NR_137051.1	*Terfezia eliocrocae*
AF276668.1	*Tirmania nivea*	MF940200.1	*Terfezia eliocrocae*
FN395015.1	*Tirmania nivea*	MF940201.1	*Terfezia eliocrocae*
JF908770.1	*Tirmania nivea*	HM056199.1	*Terfezia extremadurensis*
KJ947347.1	*Tirmania nivea*	HM056200.1	*Terfezia extremadurensis*
KJ947348.1	*Tirmania nivea*	HM056201.1	*Terfezia extremadurensis*
HM352547.1	*Tirmania pinoyi*	HM056202.1	*Terfezia extremadurensis*
HM352548.1	*Tirmania pinoyi*	HM056203.1	*Terfezia extremadurensis*
HM352549.1	*Tirmania pinoyi*	HM056204.1	*Terfezia extremadurensis*
HM352550.1	*Tirmania pinoyi*	NR_137050.1	*Terfezia extremadurensis*
MG917773.1	*Tirmania pinoyi*	HM056217.1	*Terfezia fanfani*
MH084954.1	*Tirmania pinoyi*	HM056218.1	*Terfezia fanfani*
MK478851.1	*Tirmania pinoyi*	HM056214.1	*Terfezia fanfani*
MK478852.1	*Tirmania pinoyi*	MG817381.1	*Terfezia fanfani*
MK478863.1	*Tirmania pinoyi*	KP189328.1	*Terfezia grisea*
HM056220.1	*Terfezia albida*	KP189329.1	*Terfezia grisea*
HM056221.1	*Terfezia albida*	KP189330.1	*Terfezia grisea*
NR_137053.1	*Terfezia albida*	KP189331.1	*Terfezia grisea*
HQ698098.1	*Terfezia alsheikhii*	KP189332.1	*Terfezia grisea*
HQ698099.1	*Terfezia alsheikhii*	KP189333.1	*Terfezia grisea*
HQ698100.1	*Terfezia alsheikhii*	NR_160444.1	*Terfezia grisea*
HM056207.1	*Terfezia alsheikhii*	MN512331.1	*Terfezia honrubiae*
HM056208.1	*Terfezia alsheikhii*	MN512332.1	*Terfezia honrubiae*
NR_119926.1	*Terfezia alsheikhii*	MN512333.1	*Terfezia honrubiae*

Accession No.	Species	Accession No.	Species
AF276675.1	*Terfezia arenaria*	MN512334.1	*Terfezia honrubiae*
HQ698066.1	*Terfezia arenaria*	MG818752.1	*Terfezia lusitanica*
HQ698067.1	*Terfezia arenaria*	MG818753.1	*Terfezia lusitanica*
HQ698068.1	*Terfezia arenaria*	MG818754.1	*Terfezia lusitanica*
HQ698069.1	*Terfezia arenaria*	NR_159059.1	*Terfezia lusitanica*
KF281114.1	*Terfezia arenaria*	MG640478.1	*Terfezia morenoi*
KF281115.1	*Terfezia arenaria*	MG640479.1	*Terfezia morenoi*
KP217812.1	*Terfezia arenaria*	MG640480.1	*Terfezia morenoi*
KP217813.1	*Terfezia arenaria*	MG640481.1	*Terfezia morenoi*
KP217814.1	*Terfezia arenaria*	MG640482.1	*Terfezia morenoi*
KP217816.1	*Terfezia arenaria*	MG640483.1	*Terfezia morenoi*
KP217817.1	*Terfezia arenaria*	MG640484.1	*Terfezia morenoi*
MF940176.1	*Terfezia arenaria*	MG640485.1	*Terfezia morenoi*
MF940177.1	*Terfezia arenaria*	HM056223.1	*Terfezia morenoi*
LT718226.1	*Terfezia arenaria*	NR_160498.1	*Terfezia morenoi*
LT718235.1	*Terfezia arenaria*	AF387656.1	*Terfezia olbiensis*
LT718238.1	*Terfezia arenaria*	HM056209.1	*Terfezia pini*
MF940178.1	*Terfezia boudieri*	HM056210.1	*Terfezia pini*
MF940181.1	*Terfezia boudieri*	NR_164515.1	*Terfezia pini*
MN314874.1	*Terfezia canariensis*	HM056211.1	*Terfezia pseudoleptoderma*
MN317368.1	*Terfezia canariensis*	HM056212.1	*Terfezia pseudoleptoderma*
KP728821.1	*Terfezia cistophila*	AJ272442.1	*Mattirolomyces terfezioides*
KP728823.1	*Terfezia cistophila*	AJ272443.1	*Mattirolomyces terfezioides*
KP728824.1	*Terfezia cistophila*	AJ272444.1	*Mattirolomyces terfezioides*
KP728825.1	*Terfezia cistophila*	AJ272445.1	*Mattirolomyces terfezioides*
